# Out-Of-Plane Permeability Evaluation of Carbon Fiber Preforms by Ultrasonic Wave Propagation

**DOI:** 10.3390/ma13122684

**Published:** 2020-06-12

**Authors:** Francesca Lionetto, Francesco Montagna, Alfonso Maffezzoli

**Affiliations:** Department of Engineering for Innovation, University of Salento, Via Arnesano, 73100 Lecce, Italy; francesco.montagna@unisalento.it (F.M.); alfonso.maffezzoli@unisalento.it (A.M.)

**Keywords:** ultrasound, time of flight, reinforcement, resin transfer molding (RTM), permeability, liquid composite molding, material characterization, composite manufacturing

## Abstract

Out-of-plane permeability of reinforcement preforms is of crucial importance in the infusion of large and thick composite panels, but so far, there are no standard experimental methods for its determination. In this work, an experimental set-up for the measurement of unsaturated through thickness permeability based on the ultrasonic wave propagation in pulse echo mode is presented. A single ultrasonic transducer, working both as emitter and receiver of ultrasonic waves, was used to monitor the through thickness flow front during a vacuum assisted resin infusion experiment. The set-up was tested on three thick carbon fiber preforms, obtained by stacking thermal bonding of balanced or unidirectional plies either by automated fiber placement either by hand lay-up of unidirectional plies. The ultrasonic data were used to calculate unsaturated out-of-plane permeability using Darcy’s law. The permeability results were compared with saturated out-of-plane permeability, determined by a traditional gravimetric method, and validated by some analytical models. The results demonstrated the feasibility and potential of the proposed set-up for permeability measurements thanks to its noninvasive character and the one-side access.

## 1. Introduction

By combining the attractive features of different constituents, composite materials can be properly designed for specific applications, thus replacing traditional materials in an ever-increasing variety of products and applications in aerospace, automotive, marine, nanocomposite, construction, etc. [1,2,3]. More recently, the emerging trend towards lightweight, high performance, high functionality and high sustainability components is driving the research towards nanocomposites, multi-material hybrid structures and composites derived from renewable resources or from reusing approaches [4,5,6,7,8,9]. The growing demand of polymer matrix composites in several fields has also driven the research and development of new manufacturing processes. The challenge of manufacturing high quality and complex geometry components at relatively low cost and fast cycle times has pushed toward the development of liquid composite molding (LCM) processes [10,11]. These technologies usually involve the use of dry reinforcement preforms, an assembly of dry fiber layers that have been pre-shaped to the form of the desired product and bonded together using a binder resin, which are impregnated by resin injection or infusion into a mold [12,13]. Autoclave curing is usually used to limit the final void content, which remains a significant issue, related to the filling process, fiber wetting and tows impregnation. To achieve the required mechanical performance of the composite parts, multi-layered preforms with different architecture are generally used, such as unidirectional non crimped fabric and plain or twill woven fabric [14]. Furthermore, LCM requires low viscosity resins, which upon crosslinking would be brittle, being made of low molecular weight oligomers. Therefore, the higher molecular weight oligomers of the resin formulation, solid at room temperature, are added to the reinforcement as fiber coating or powders that are soluble in the matrix. These oligomers play also a role in preform fabrication, acting as low melting tackifying agent able to bind a ply to another upon heating [15]. The required performances are achieved in the final component only if a fast and complete filling of the preform with liquid resin is obtained thanks to the optimization of LCM process [16,17]. A suitable process design is necessary, which requires knowledge about different parameters affecting the filling behavior, such as mold geometry, resin viscosity, number and position of inlet and vent ports, etc. [18]. Besides the previous parameters, one of the most critical is the permeability of the reinforcement, defined as the resistance exhibited by the fibrous reinforcement against the resin flow [19]. This property determines the impregnation of the textile reinforcement with liquid resin and strongly depends on the reinforcement architecture, fiber orientation and volume fraction and on the procedure adopted to assemble and stabilize the plies into the preform [20]. The variability of textiles and the preforming operations affect the permeability of the fibrous reinforcements, resulting in possible unexpected or unwanted resin flow patterns [21]. Generally, permeability data are not available from the fabric manufacturer and must be determined depending on the fiber volume fraction. An accurate characterization of the reinforcement permeability is therefore fundamental to estimate the optimum process parameters for manufacturing high-quality components. 

The permeability, K, of porous media is defined by Darcy’s law [22] correlating the flow velocity V, pressure drop, ΔP, and viscosity, η, in a unidirectional flow over the length of the porous medium L:

(1) $$V=-\frac{K}{\eta }\frac{\Delta P}{L}$$

The permeability of unidirectional preforms is an anisotropic property and is described by a second-order tensor [20]. Generally, by accounting for symmetry and assuming no coupling between in- and out-of-plane flow, only three components of the permeability tensor are not zero: (i) longitudinal in-plane permeability K_1_, where in-plane refers to the reinforcement ply; (ii) transversal in-plane permeability K_2_, oriented perpendicular to K_1_ and lower than K_1_; (iii) out-of-plane permeability K_3_, oriented perpendicular to K_1_ and K_2_, lower than K_1_ and of the same order of K_2_ [21].

Different measurement methods of permeability are available in the literature [20,23,24]; each is capable to measure saturated or unsaturated permeability. Saturated permeability is a steady state permeability measured under a constant flow rate when the fiber reinforcement is fully saturated by the test fluid. Unsaturated permeability, also named unsteady-state or transient permeability, is measured when the reinforcement in the mold is progressively impregnated by a test fluid injected under a constant pressure [25]. However, there is a complete lack of standardization in the methods and experimental set-up, and the data, obtained using different methods and different fabric architecture, are not consistent [25]. Some benchmark studies demonstrated that when the same measuring procedure is used, the results are very different if different parameters are used, such as in terms of injection pressure and test fluid [26]. Most studies have been focused only on the in-plane permeability [27,28,29], disregarding the out-of-plane permeability, which is more difficult to measure accurately [30]. However, the out-of-plane (or through thickness) permeability is the dominant property in the infusion of large and flat panels with a high thickness when a resin distribution net is used. Therefore, its determination is of fundamental importance for the efficiency and robustness of LCM processes. The traditional video recording methods used for monitor flow front in RTM processes and determining in-plane permeability, cannot work well in the case of through-thickness permeability [31].

Several nondestructive methods have been investigated based on thermocouples [32], on the reflection at the flow front of electrical signals from thin metallic wires inside the fibers [33] guided mechanical waves [34], piezoelectric sensors [35] dielectric sensors [36] embedded in the fibers and pressure sensors positioned in the mold [37,38]. All of these measurement methods require both direct contact with the resin in order to detect the flow-front position or complete mold-filling and sensor embedded in the preform or into the mold [39,40]. The sensor embedding is not always allowed and is hence limited in application. Mounting sensors into the mold can affect the vacuum tightness of the mold and the sensors usually leave marks on the part surface so they can be only located at the border but not in the areas of interest [39].

Ultrasonic wave propagation has been widely recognized as a nondestructive testing (NDT) method applied to for the estimation of the physical and mechanical properties or the damage composite materials [41,42,43,44,45] and for process monitoring of composite materials [46,47]. Even the application of high intensity ultrasound in composite processing and joining present the potential for online monitoring [48,49,50]. Some research works proved the feasibility of using ultrasonic imaging systems for flow front monitoring even if the technique was limited to a single ply or thin preforms due to the severe attenuation of the ultrasound waves [31,51]. Stoven et al. [52] developed a set-up based on two ultrasonic probes operating in through transmission mode for flow monitoring and permeability determination in thickness direction. The ultrasonic based method presents several advantages: i) absence of any direct contact between the transducer and the composite and of any embedded sensor as the sound waves can be send through the mold wall; ii) low cost; iii) no disturbance to the fiber stack and liquid flow; iv) measurement of unsaturated permeability; v) reduced effort for preparing the experimental set-up; vi) no need of transparent tools; vii) compatible with all the fiber typologies [10,39,53].

Despite the numerous advantages, these ultrasonic based methods are still at a laboratory level due to the experience required to manage weak acoustic signals and the limitation of the resolution depth in thick preforms [53,54]. Moreover, the ultrasonic set-up described in the literature works in through transmission method with two ultrasonic transducers acting one as emitter and the other as receiver of ultrasonic waves [52], but sometimes the two-side access to the composite part is very difficult to achieve. 

The aim of this work is to present a new experimental set-up for the measurement of unsaturated through thickness permeability based on the ultrasonic wave propagation in pulse echo mode, i.e., by using only one ultrasonic transducer working both as emitter and receiver of ultrasonic waves. Compared to the ultrasonic based systems previously reported in the literature, the proposed set-up enables one-side access, which is of great importance in composite manufacturing. This work stands out of the few previous related papers [10,52,53] by using the pulse echo technique with a single transducer, working both as emitter and receiver of ultrasonic waves, positioned at one side of the preform. The literature studies, in fact, use the ultrasonic transmission method, which is not always feasible when the composite is not accessible from both sides. The single ultrasonic probe, coupled to a vacuum assisted resin infusion (VARI) system, allows in-situ monitoring of unsteady preform impregnation. The system has been applied to multi-layered carbon fiber preforms. The robustness of the system has been tested on three different carbon fiber preforms used in aerospace field, each obtained by stacking balanced or unidirectional plies and then tested in vacuum infusion experiments. Saturated out-of-plane permeability has also been measured by a traditional gravimetric method and the results obtained by the two techniques have been compared. Finally, another novelty element is the validation of the permeability data, calculated from ultrasonic measurements, by some analytical models.

## 2. Materials and Methods

### 2.1. Materials

**Figure 1:**
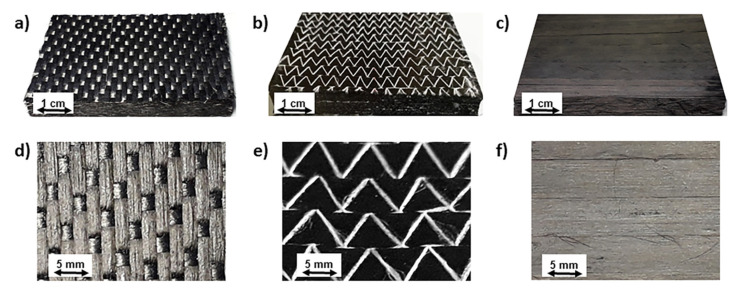
Preforms for permeability measurements: (**a**,**d**) balanced, (**b**,**e**) unidirectional (UD) stitched and (**c**,**f**) unidirectional (UD) consolidated by AFP.

The preforms for permeability measurements were obtained using balanced and unidirectional carbon fiber fabrics and unidirectional (UD) tapes. The preforms investigated in this work are shown in Figure 1 and their properties are reported in Table 1.

Preform A was a balanced fabric, produced by Hexcel (Stanford, CA, USA) with the trade name G0926 HS06K. It was a 0.38-mm-thick 5H satin with HEXTOW AS4C GP 6K with a nominal weight of 375 g/m^2^, the same weight distribution in warp and weft directions and filled with an epoxy binder. The binder content was 4% of the weight of the carbon fiber [15]. Preforms A were obtained in laboratory by vacuum bagging of [0]_22_ stacks of G0926 5H satin fabric in an oven during the following thermal cycle: heating up to 100 °C at 3 °C/min, isotherm for 75 min at 110 °C and cooling to room temperature at 1.5 °C/min. This cycle was able to dissolve the epoxy powders used as binder and to give stiffness to the preforms.

Preform B was a stitched unidirectional carbon fabric, produced by Solvay S.A. (Bruxelles, Belgium) with the trade name BNFC-24k IMS-(0)-196-600, obtained with IM65 carbon fibers containing a binder for preform fabrication. Preforms A and B, were provided by Leonardo SpA (Foggia, Italy), according to the same consolidation cycle.

Preform C was made of an unidirectional TX 1100 IMS65 24 k fabric, produced by Solvay S.A. (Bruxelles, Belgium) with IM65 carbon fibers. Preforms C, provided by Novotech Aerospace Advanced Technology srl (Avetrana, Italy), were prepared by automated fiber placement (AFP) using a tape width of 8 mm.

Permeability experiments were performed at room temperature using polyethylene glycol 400 (PEG400) as test fluid provided by Sigma-Aldrich (Milano, Italy). This latter has been chosen since its viscosity value at room temperature is in the viscosity range of a thermosetting resin used in resin infusion processes at high temperature. The viscosity of the test fluid was characterized by rheological analysis carried out in an ARES parallel plate rheometer with a 50-mm plate diameter (Rheometric Scientific).

### 2.2. Experimental Set-Up for Permeability Measurements by Ultrasonic Wave Propagation

**Figure 2:**
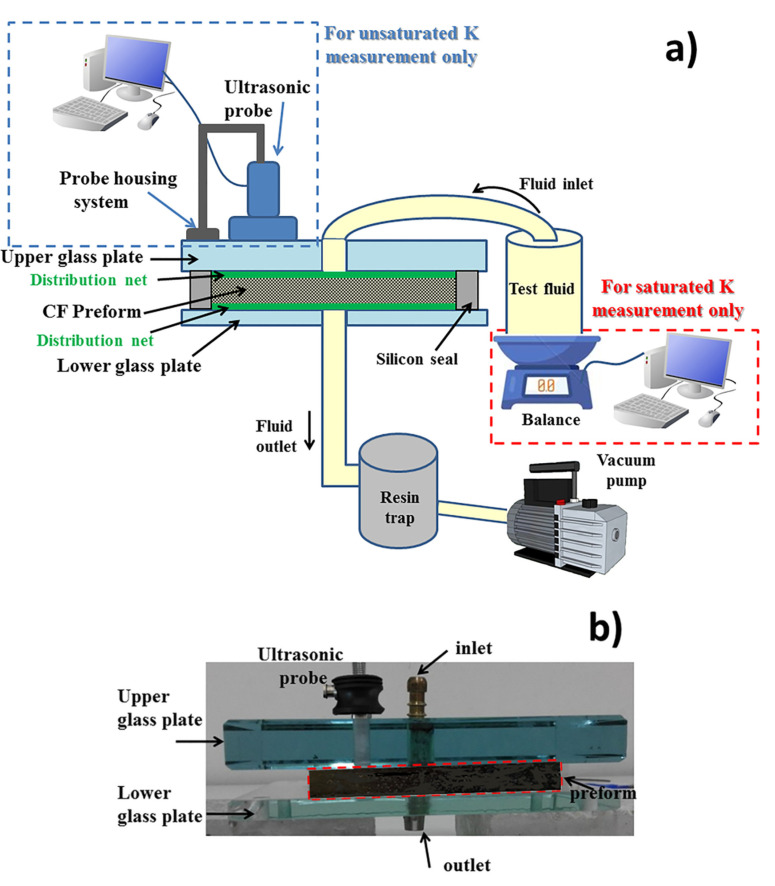
Experimental set-up for out-of-plane permeability measurements by ultrasonic wave propagation: (**a**) general sketch not in scale; (**b**) particular of the ultrasonic measurement device.

The experimental set-up for out-of-plane permeability measurements by ultrasonic wave propagation is sketched not in scale in Figure 2. The carbon fiber (CF) preform was placed between two glass plates, each of them containing a hole for the inlet and outlet of the impregnation fluid. The thickness of the glass plates was 8 mm and 20 mm for the lower and upper plate, respectively. The higher thickness was chosen in order to avoid overlapping echoes due to the reflections from the glass plate and the wet carbon fiber preform. In order to provide the vacuum tightness of the measurement system, a silicone seal was used. A resin distribution net at the top and bottom side of the sample allowed a through-thickness unidirectional flow under a constant injection pressure, obtained by a Vacuum Assisted Resin Infusion (VARI). Fiber volume fraction was changed by adjusting the distance between the glass plates. After turning on the vacuum pump, the inlet valve was opened leading the fluid to flow from the tank through the preform. Since the measurement was carried out in unsteady conditions, an unsaturated permeability was determined. During the ultrasonic measurement, the balance—framed by a red dotted line in Figure 2—was not used.

An ultrasonic transducer (A109-R Olympus Italia srl, Milano, Italy, center frequency =5 MHz, active diameter =13 mm) was fixed on the upper glass plate by a probe housing system capable to keep a constant force on the transducer. The ultrasonic frequency has been chosen as the best compromise among the wave attenuation and resolution, which increase with frequency, and the echo damping, which decreases with frequency and can lead to the overlapping of echoes related to different interfaces. An ultrasonic couplant gel was used to provide the transmission of sound energy between the transducer and the glass plate. The ultrasonic transducer, connected with a pulser-receiver (Sofratest, Ecquevilly, France), worked in pulse-echo mode, acting as emitter and receiver of ultrasonic waves at the same time [55]. The transducer was excited by a broadband pulser with an electric voltage of 200 V and a pulse duration of 200 nanoseconds. The echoes, sampled at a frequency of 60 MHz, were composed of 2048 points. The received signals, after digitalization, were automatically recorded each 0.15 sand displayed by a custom made software. Ultrasonic waves were continuously sent through the preform and it was possible to measure their time-of-flight that is the time taken for the wave to pass the preform and come back to the transducer after reflection at interfaces characterized by different acoustic impedances [56]. The two first round-trip echoes in the wet preform were considered. The ultrasonic measurement device has been set-up for carbon fiber preforms up to 10 mm. However, thicker preforms can be investigated by increasing the thickness of the upper glass plate.

### 2.3. Experimental Set-Up for Measurement of Saturatedpermeability

The same experimental set-up sketched in Figure 2 was used also for the measurement of saturated permeability. In this case, the ultrasonic probe was not used since a gravimetric measurement method was adopted. A becher filled with the test fluid was placed on a balance and, after turning on the vacuum pump, the inlet valve was opened leading the fluid to flow through the preform. Out-of-plane saturated permeability was measured after achieving steady state conditions. When the preform was saturated with the fluid, the quantity of fluid that entered from the inlet was equal to the quantity that came out from the outlet. A constant flow rate, measured by gravimetric method, under a constant pressure generated by vacuum was produced across the thickness of the different preforms. During the test, the weight of the fluid was measured as a function of time using a balance with a precision of 10^−4^ g.

## 3. Results

### 3.1. Rheological Analysis of the Model Fluid

**Figure 3:**
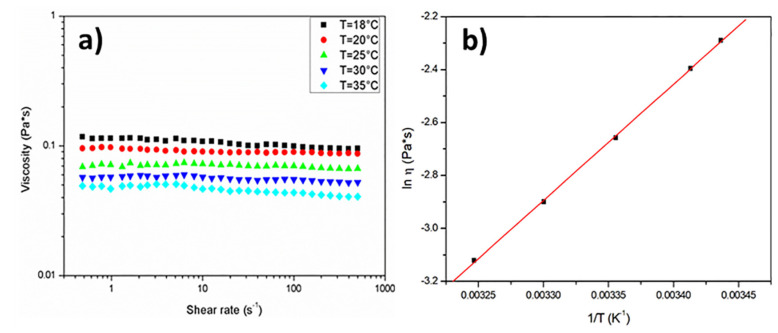
(**a**) Effect of the shear rate on the viscosity of the test fluid at different temperatures; (**b**) temperature dependence of the viscosity of the test fluid.

As shown in Figure 3a, PEG400 presents a Newtonian behavior, indicating that its viscosity is not dependent on the shear rate. The temperature dependence of the viscosity has been modeled by an Arrhenius like equation, a common approach for oligomers and polymers at a temperature much higher than their T_g_ [57]:

(2) $$\eta =\eta_{0}exp\left(\frac{E_{a}}{RT}\right)$$

where η_0_ is a pre-exponential factor, T the absolute temperature, R the universal gas constant and E_a_ the activation energy. The nonlinear fitting of PEG 400 viscosity with Equation (2) (see Figure 3b) provides an E_a_ value of 36.9 kJ/mol. Depending on the temperature of the infusion, Equation (2) has been used to determine the value of viscosity needed for permeability calculation from Darcy’s law.

### 3.2. Saturated Out-Of-PlanePermeability Measurements by VARI Process 

**Figure 4:**
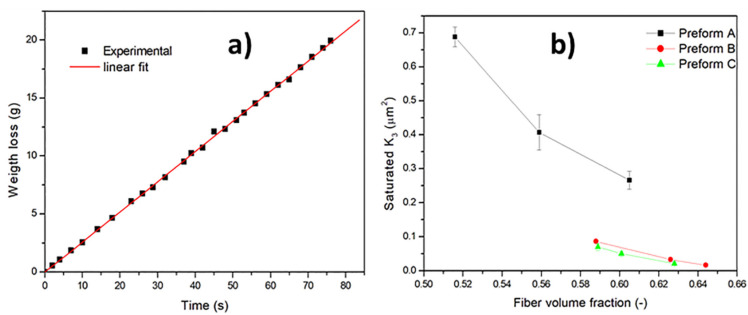
(**a**) Weight loss as a function of time for preform A; (**b**) saturated out-of-plane permeability as a function of fiber volume fraction.

Saturated out-of-plane permeability has been determined from gravimetric measurements at constant flow rate during a Vacuum Assisted Resin Infusion (VARI) process [20]. When steady state conditions are achieved, the quantity of the fluid that enters the preform is equal to the quantity that comes out per time unit. The decrease in weight of the fluid is then monitored as a function of the time. The out-of-plane saturated permeability K_3-sat_ is thus obtained by plotting the fluid weight as a function of time, t, according to the following equation:

(3) $$QAt=Weight=\frac{\rho K_{3-sat}A}{\eta }\frac{\Delta P}{L}t$$

where Q is the flow rate, A is the flow channel cross-sectional area, ρ is the fluid density, ΔP is the pressure difference (the pressure drop in the pipes was neglected, as well as the effect of gravity), L is the specimen thickness, respectively. The fluid viscosity η has been determined according to Equation (2), accounting for the test temperature. A typical plot of the weight loss as a function of time is reported in Figure 4a for Preform A.

Considering that the preform is placed between two glass plates at a cavity height h, the fiber volume fraction of the preform can be obtained according to following equation:

(4) $$V_{f}=\frac{nS_{0}}{\rho_{f}h}$$

where n is the number of plies, ρ_f_ is the fiber density and S_0_ the areal weight of a single ply.

The effect of fiber volume fraction on the saturated out-of-plane permeability of all the investigated preforms is reported in Figure 4b. The values are the average of at least three measurements. As expected, by increasing the fiber volume fraction, K_3_ decreases. The same reinforcement can be characterized by a different permeability depending on the fiber content, related to the cavity height. The different K_3_ values among the different preform typologies can be attributed to the different structure of the materials. Preform A is a woven UD fabric with stabilizing weft tows, while the Preform B and C are unidirectional, the former stabilized by stitches, the latter, used in AFP, is a true UD tape. Comparing preforms A and B, both obtained by vacuum bagging, the same pressure produced a higher compaction of fibers for preform B than for preform A, thus leading to a higher fiber volume fraction. The dependence of permeability on fiber volume fraction is different for preform A since it is more compressible than the other two as a consequence of the presence of a woven fabric in it.

The measured saturated out-of-plane permeabilities are also reported in Table 2. To the best of author knowledge, a comparison with literature data can be only made between the values of unidirectional Preform C and the work of Aziz et al. [58] who measured K_3-sat_ by a saturated unidirectional flow device on TX 1100 quasi-isotropic preforms produced by automated fiber placement with different band widths. In particular, at a fiber content of 58%, the obtained saturated permeability was 0.0831 µm^2^ and 0.0185 µm^2^ on preforms with a band width of 6.35 mm and 12.7 mm, respectively. The experimental value of 0.702 µm^2^ obtained in this study on Preform C (produced by automated fiber placement with a band width of 8 mm) at V_f_ = 58.8% is in the range measured by Aziz et al. [58], at the same preform material and fiber content, even if the preform architecture is different.

### 3.3. Out-Of-PlanePermeability Measurements by Ultrasonic Wave Propagation

Unsaturated K_3_ permeability has been measured using a single ultrasonic transducer working in pulse-echo mode, i.e., working either as emitter either as receiver. A dedicated software is able to visualize and save the echoes and to record the time delay between them (time of flight). The principle underlying the measurement is based on the reflection of the ultrasonic wave at the interface between two materials of different density and elastic properties [59]. Since ultrasounds are almost completely reflected at the interface between a solid or a liquid and air, and strongly attenuated by scattering in porous media, the reflected ultrasonic signal (echo) from a dry preform is nearly negligible while that one from a wetted preform is clearly detectable.

**Figure 5:**
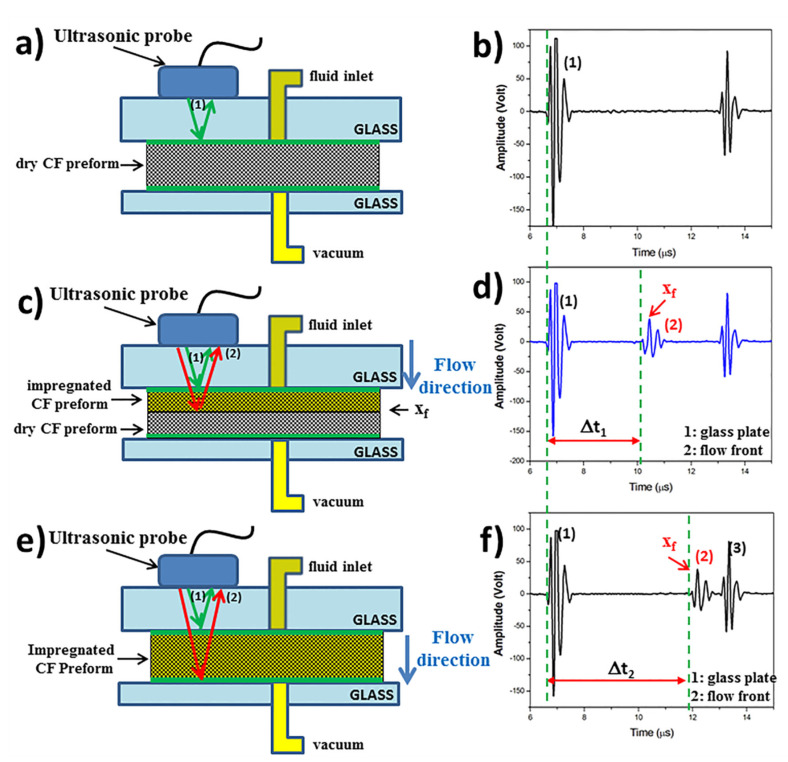
Ultrasonic wave path and corresponding echograms at different stages of the impregnation process: (**a**,**b**) no impregnation; (**c**,**d**) partial impregnation;(**e**,**f**) complete impregnation.

As sketched in Figure 5a, when the infusion has not yet begun and the carbon fiber (CF) preform is still dry, the ultrasonic wave is almost completely reflected at the glass/preform interface because ultrasound is very scarcely transmitted in air. Therefore, in Figure 5a, only one ultrasonic wave path is sketched by green arrows. The corresponding echogram (Figure 5b) reports the signals relative to the multiple reflections back and forth from the upper glass plate. No echo relative to reflections from the preform is observed.

As sketched in Figure 5c, after the beginning of infusion, when the preform is partially impregnated, a fraction of the ultrasonic wave is transmitted at the glass/wet preform interface and travels through the wet preform but it is totally reflected (red arrows) at the flow front, i.e., at the interface between the wet preform and the dry preform. The signals relative to the reflections at the glass/wet preform interface (echo No.1) and at the wet/dry preform interface (echo No.2) are shown in Figure 5d. The time difference, Δt, between the two peak times relative to the echo from the partial impregnated preform (No.2) and the echo from the glass plate (No.1) is called time of flight (TOF)and corresponds to the time the ultrasonic waves take to travel twice between the glass plate and the impregnation front.

When the preform is filled, the path of ultrasonic wave is represented by the red arrows in Figure 5e. Consequently, the echo No.2 in Figure 5f is right shifted while the position of the two echoes No.1 and No.3 relative to multiple reflections at the top glass plate-preform interface remains constant. Therefore, the recorded time of flight of the wave reflected from preform increases since the distance of the flow front from the transducer increases. As observable from Figure 5d–f, the TOF of partial impregnated preform Δt_1_ is lower than that of completely impregnated preform Δt_2_. The echo No.2 can be therefore used to monitor the flow front position through the thickness of the preform.

**Figure 6:**
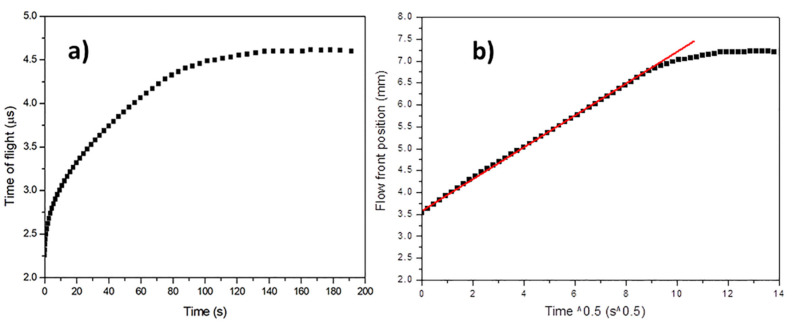
Preform C at fiber volume fraction of 0.628: (**a**) measured ultrasonic time of flight during the infusion; (**b**) calculated flow front position from ultrasonic data.

A custom-made software has been used to record the TOF during the infusion of the carbon fiber preform. The time of flight measured during an infusion experiment is reported as a function of the infusion time in Figure 6a. TOF increases when the infusion starts, then it reaches a plateau value when the preform is fully impregnated. At this point, it can be deduced that the impregnation of the preform is complete.

The TOF value can be used to determine the distance x_f_ of the flow front from the glass plate, which in the pulse echo mode is given by:

(5) $$x_{f}=\frac{\Delta tc}{2}$$

where Δt is the TOF value and c the longitudinal wave velocity in the wet preform. This latter has been determined for each analyzed preform at each fiber volume content, by applying the inverse rule of mixture for a composite material with carbon fibers and PEG400 matrix:

(6) $$c_{}=\frac{1}{\frac{V_{f}}{c_{f}}+\frac{V_{m}}{c_{m}}}$$

where V_f_ and V_m_ are the volume fraction of carbon fiber and PEG matrix, respectively, while c_f_ and c_m_ are the sound velocity of carbon fiber and PEG matrix, respectively. The sound velocity of PEG 400 has been measured and it is equal to 1507 m/s. The sound velocity of carbon fiber has been estimated from the elastic modulus and density of the different fibers used for the three preforms according to the following equation:

(7) $$c_{f}=\sqrt{\frac{E}{\rho }}$$

where ρ is the density, equal to 1.78 g/cm^3^ for all the fibers, while the elastic modulus E is equal to 231 GPa for the carbon fibers of preform A and 290 GPa for those of preform B and C (see Table 1). A sound speed of 11,392 m/s for carbon fibers of preform A and 12,764 m/s for carbon fibers of preform B and C has been obtained from Equation (7). The ultrasonic velocity value c, calculated according to Equation (6), is considered constant during the experiment. The eventual presence of microscopic air bubbles during the flow does not affect ultrasonic velocity but can decreases the amplitude of the ultrasonic wave [46].

The x_f_ values as a function of square root of time, reported in Figure 6b, present an initial linear behavior which can be used for the determination of unsaturated out-of-plane permeability K_3-unsat_ of the preform using the Darcy’s equation, valid in the case of one-dimensional flow and constant injection pressure:

(8) $$x_{f}=\sqrt{\frac{2K_{3-unsat}{}_{}\Delta P}{\eta }}\sqrt{t_{f}}$$

where ΔP is the pressure difference and η the viscosity of the fluid, determined according to Equation (2), accounting for the test temperature.

The slope obtained from the linear fit of the data can be used for the determination of K_3-unsat_, according to the following equation:

(9) $$K_{3-unsat}=\frac{{\left(slope\right)}^{2}\eta }{2\Delta P}$$

The values of saturated and unsaturated permeabilities of all the investigated preforms are reported in Table 2. The values are the average of at least three measurements. For each preform, as the fiber volume fraction increases, the permeability values decrease, due to the increased fiber compaction that leads to a reduced flow rate under the same pressure gradient. Moreover, at the same fiber volume fraction and for the same preform, the saturated out-of-plane permeability is higher than the unsaturated out-of-plane permeability. The discrepancy between saturated and unsaturated permeabilities, which has been observed also for in-plane permeability, has been explained by many authors by the void formation during the unsaturated flow. During the filling process, the liquid flow advancement is not uniform and air is entrapped at the flow front, creating partially impregnated zone leading to a change of the hydraulic conductivity [25].

**Figure 7:**
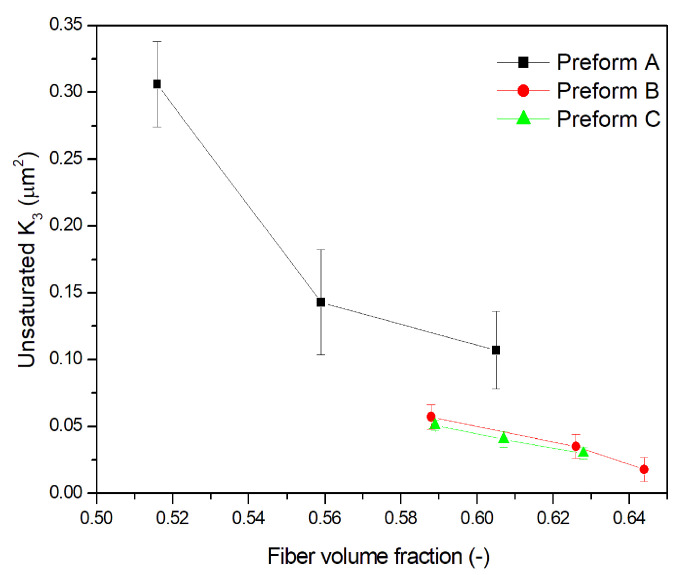
Unsaturated out-of-plane permeability as a function of fiber volume fraction.

The effect of fiber volume fraction on the unsaturated out-of-plane permeability of all the investigated preforms is reported in Figure 7. Preform A is characterized by higher permeability at similar V_f_, between 0.585 and 0.605, probably as a consequence of the different architecture of each ply. The weft tows in preform A, not only limit its compressibility leading to a lower V_f_ at the same compaction level but are also responsible of the presence of lower resistance pathways to fluid advancement. On the other hand, lower permeability of preforms B and C at the same V_f_, can be attributed to the absence of low resistance pathways. The slightly higher permeability of preform B compared to C is probably due to the stitching yarns used in preform B to stabilize the UD arrangement (Figure 1). The impregnation experiments made for the measurement of in-plane permeabilities K_1_ and K_2_ (not reported), indicated that these yarns are more easily wetted by the fluid which find a low resistance pathways inside them [60]. On the other hand, the carbon fiber tows of preform C, obtained using tapes stabilized by a binder also acting as tackifying agent during the lay up, are better compacted, even if the control of temperature and pressure applied during AFP is less accurate than in vacuum bagging.

A comparison with literature data is very difficult since the reported permeability data are characterized by a strong scattering depending on the measurement method, the fiber preform, the test fluid and the fluid injection parameters. As also observed also by Konstantopoulos et al. [53], the literature data can be used only as a reference to confirm that the results of this study lie in the correct order of magnitude: the unsaturated data obtained by Agougue et al. [30], on TX100 preform at V_f_=58% are very close to the results obtained in this work. Despite the different preform architecture (quasi-isotropic for [30] and unidirectional in this study) and the different measurement method, the same order of magnitude of 100 µm^2^ is obtained by correcting the value reported by Agogue et al. [30] by the (1-V_f_) coefficient.

### 3.4. Mathematical Modeling

The values of out-of-plane permeability, both saturated and unsaturated, of the three different preforms have been further validated by comparison with the results of predictive models. As recently reported by Karaki et al. [18,61], among the several mathematical models available in the literature for estimating the permeability of unidirectional reinforcements, the most accurate ones, able to fit the experimental data of out-of-plane permeability of unidirectional yarns, are those proposed by Gebart [62] and Berdichevsky and Cai [63].

Gebart [62] derived an analytical model to predict the unidirectional permeability starting from Navier-Stokes equation, considering two fibers arrangements, a quadratic array and a hexagonal array:

(10) $$K_{3}{=C}_{1}{\left(\sqrt{\frac{V_{\mathrm{fmax}}}{V_{f}}}-1\right)}^{\frac{5}{2}}r_{f}^{2}$$

where V_f_ is the fiber volume fraction, r_f_ the fiber radius and the constants C_1_ and V_fmax_ depend on the fiber packing arrangement according to the values reported in Table 3.

Berdichesvky and Cai [63] derived an unified empirical model where Stokes flow and Darcy flow are considered at different regions and the gap between neighboring fibers governs the flow resistance:

(11) $$K_{3}={0.229r}^{2}\left(\frac{1.814}{V_{a}}-1\right){\left(\frac{\left(1-\sqrt{\frac{V_{f}}{V_{a}}}\right)}{\sqrt{\frac{V_{f}}{V_{a}}}}\right)}^{2.5}$$

where V_f_ is the fiber volume fraction and V_a_ is equal to 0.9069 and 0.7854 in case of hexagonal or quadratic fiber arrangement, respectively.

**Figure 8:**
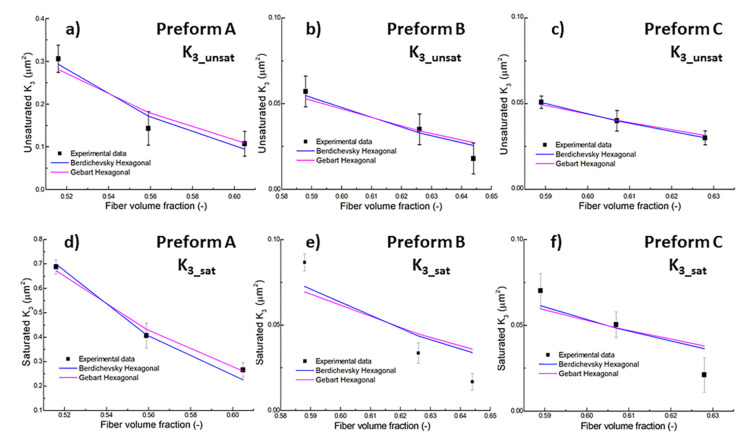
Mathematical modeling of unsaturated (**a**–**c**) and saturated permeability (**d**–**f**) values: (**a**,**d**) preform A; (**b**,**e**) preform B; (**c**,**f**) preform C.

Models predictions and the experimental results on the unsaturated and saturated permeabilities are compared in Figure 8, where only the best fit among modeling and experimental data are reported. A nonlinear fit of the K_3_ values as a function of fiber volume fraction has been performed using Equations (10) and (11), in order to obtain the fiber radius r_f_, whose values are reported in Table 4. The quadratic fiber arrangement is not able to properly model the experimental values, as inferred by the high differences reported in Table 4, since the obtained fiber radius is higher than the nominal value, which is equal to 3.35 μm for Preform A and 2.5 μm for Preforms B and C. Therefore, the fit obtained with the quadratic fiber arrangement with both the models are not reported in Figure 8 for the sake of clarity.

On the other hand, the best fit is always obtained adopting the hexagonal array and Berdichevsky and Cai model, with differences from the nominal fiber radius lower than 15%. Only the best fit of saturated K_3_ of preform A, with all models, leads always to fiber radii much larger than the nominal ones. From Figure 8 it can be observed good agreement among the unsaturated K_3_ values calculated by ultrasonic measurement and the model prediction. This can be considered a further validation of the proposed measurement method, based on ultrasonic wave propagation.

It should be also underlined that the schematization in hexagonal and quadratic array is often ideal since the fiber distribution is often irregular and falls in between hexagonal and quadratic arrangement. Fiber clustering, in real preforms, makes reasonable the almost always higher radii obtained from fitting compared to the nominal ones.

## 4. Conclusions

An experimental set-up for the measurement of unsaturated through thickness permeability based on the ultrasonic wave propagation in pulse echo mode was proposed. A single ultrasonic transducer, working both as emitter and receiver of ultrasonic waves, was used to monitor the through thickness flow front during a vacuum assisted resin infusion set-up. The new measurement method was tested on three thick carbon fiber preforms, obtained by stacking and vacuum bagging or by automated fiber placement of unidirectional plies. The ultrasonic data were used to calculate unsaturated out-of-plane permeability applying Darcy’s law. The permeability results were compared with saturated out-of-plane permeability, determined by a traditional gravimetric method.

For each preform, as the fiber volume fraction increased, the permeability values decreased, due to the increased fiber compaction that lead to a slowdown of the fluid flow. Moreover, at the same fiber volume fraction and for the same preform material, the saturated out-of-plane permeabilities were higher than the unsaturated out-of-plane permeabilities.

Finally, the permeability data were validated by two analytical models: Berdichevsky and Cai model and Gebart model. The best agreement was obtained using Berdichevsky and Cai model assuming a hexagonal fiber arrangement.

## Figures and Tables

**Table 1:** Carbon fiber preform configuration.

	A Balanced Preform	B Stitched Preform	C AFP Preform
Carbon fiber type	G0926 HS06K (Hexcel)	BNCF-24KIMS-(0)-196-600 (Cytec)	TX 1100 IMS65 24k(Cytec)
Fiber diameter (μm)	6.9	5.0	5.0
Fiber elastic modulus (GPa)	231	290	290
Fiber areal weight (g/m^2^)	375	196	200
Number of layers	22	40	40
Nominal preform size (mm^3^)	80 × 50 × 8	80 × 50 × 8	80 × 50 × 8
Preform manufacturing process	vacuum bagging	vacuum bagging	automated fiber placement

**Table 2:** Out-of-plane saturated and unsaturated permeabilities for the investigated preforms.

Material	V_f_ (%)	Out-Of-Plane Saturated Permeability (µm^2^)	Out-Of-Plane Unsaturated Permeability (µm^2^)
Preform A	51.6	0.688 ± 0.0290	0.306 ± 0.032
	55.9	0.407 ± 0.0052	0.143 ± 0.039
	60.5	0.266 ± 0.0027	0.107 ±0.029
Preform B	58.8	0.087 ± 0.005	0.057 ± 0.009
	62.6	0.034 ± 0.006	0.035 ± 0.009
	64.4	0.017 ± 0.005	0.018 ± 0.009
Preform C	58.9	0.070 ± 0.010	0.051 ± 0.003
	60.1	0.050 ± 0.007	0.040 ± 0.006
	62.8	0.021 ± 0.005	0.029 ± 0.004

**Table 3:** Gebart model: constant values for quadratic and hexagonal fiber arrangement.

Fiber Arrangement	C_1_	V_fmax_
Quadratic	$\frac{16}{9\pi \times \sqrt{2}}$	$\frac{\pi }{4}$
Hexagonal	$\frac{16}{9\pi \times \sqrt{6}}$	$\frac{\pi }{2\times \sqrt{3}}$

**Table 4:** Unsaturated out-of-plane fiber radius obtained from the mathematical modeling of the experimental K_3_ values and percentage difference from the nominal fiber radius.

Material	Out-Of-Plane Permeability	Model	Fiber Radius from Model Best Fit, r_f_ (µm)	Nominal Fiber Radius r_fn_ (µm)	Difference [(r_fn_ − r_f_)/r_f_] × 100 (%)
Preform A	Unsaturated	Gebart-hexagonal	4.57	3.45	+32
		Gebart-quadratic	5.45	3.45	+58
		Berdichevsky-hexagonal	3.23	3.45	−6.8
		Berdichevsky-quadratic	4.80	3.45	+39
	Saturated	Gebart-hexagonal	7.09	3.45	+105
		Gebart-quadratic	8.41	3.45	+144
		Berdichevsky-hexagonal	4.99	3.45	+45
		Berdichevsky-quadratic	7.39	3.45	+114
Preform B	Unsaturated	Gebart-hexagonal	2.89	2.5	+16
		Gebart-quadratic	4.04	2.5	+62
		Berdichevsky-hexagonal	2.20	2.5	−12
		Berdichevsky-quadratic	3.73	2.5	+49
	Saturated	Gebart-hexagonal	3.32	2.5	+33
		Gebart-quadratic	4.69	2.5	+88
		Berdichevsky-hexagonal	2.53	2.5	+1.2
		Berdichevsky-quadratic	4.43	2.5	+77
Preform C	Unsaturated	Gebart-hexagonal	2.81	2.5	+12
		Gebart-quadratic	3.87	2.5	+55
		Berdichevsky-hexagonal	2.13	2.5	−15
		Berdichevsky-quadratic	3.65	2.5	+46
	Saturated	Gebart-hexagonal	3.09	2.5	+24
		Gebart-quadratic	4.29	2.5	+72
		Berdichevsky-hexagonal	2.35	2.5	−6
		Berdichevsky-quadratic	4.05	2.5	+62
